# Notes and News

**Published:** 1897-05-22

**Authors:** 


### Notes and News.

(Collected and Communicated.)

HOSPITAL MEETINGS.

Hospital for Sick Children, Great Ormond Street.—Annual General Court, Wednesday, May 26th, 4.30 p.m., in Board Room of the Hospital.

The Earl of Erroll will preside at the Annual Meeting of the Children's Country Holiday Fund, on Monday, May 24th, at the Royal United Service Institution, Whitehall.

---

Lord Lister presided at the dinner of the Edinburgh University Club, last week, held at the Holborn Restaurant.

---

The so-called Optometry Bill, granting opticians the right to refract eyes and fit glasses, which has been before the New York Legislature, was defeated by a vote of 73 to 51.

---

The plague reports are greatly improving, but there are districts in which it is still markedly prevalent. The Cutch district to the north of Bombay still holds a bad record.

---

At the recent terrible fire in Paris the efficiency of the Urban Ambulance Service was put to a strong test, and its excellence fully demonstrated. The carriages were promptly requisitioned, and their use helped to minimise the horror of the removal of the remains.

---

Extensive improvements have lately been effected in the operating theatre of the Prince Alfred Hospital, Sydney, at a cost of nearly £1,000. The theatre was reopened on March 15th, and is now splendidly equipped for the large amount of work done in it. The lighting has been improved by the addition of windows in the roof, the area for the operating surgeons and their assistants has been walled round by marble slabs, the floor retiled, and the wash-basins fitted with foot-taps. The water system has also been renovated, the cold water being all sterilised, drawn from a copper tank into which boiling water is passed from the hot-water system, the tank being also provided with a steam-pipe and worm of thirteen coils to reboil the water if necessary. The vent-pipe from the tank ends in a copper chamber packed with sterilised wool. Radiators have been fitted up for heating purposes.

Mrs. Aitkin, of Holcome Hall, Ramsbottom, has presented a cottage hospital, to contain nine beds, to the locality, in commemoration of Her Majesty's long reign.

---

The Earl of Jersey will preside at the meeting of the National Refuges and Training Ships, *Arethusa* and *Chichester*, at Exeter Hall, on Tuesday, 25th inst., at half-past five. A choir of 500 young people, including sailor boys, will sing.

---

The "Tiny Tim" Cot has been endowed at Guy's Hospital, in memory of Charles Dickens. The idea originated with Dr. Knott, of Portsmouth. The bulk of the money was raised at Portsmouth itself, which was Dickens's birth-place.

---

The new Convalescent Home of the Friendly Societies of the North-Eastern Counties has been opened at Grange-over-Sands by Sir John Hibbert. We published plans and description of the building in our issue of April 10th last. The cost of the Home is about £3,000.

---

The Augustus Harris Memorial Committee have made a donation of £1,000 to Charing Cross Hospital for the endowment of a bed in perpetuity to the memory of Sir Augustus Harris. The bed is to be reserved for patients connected with the dramatic and music-hall professions.

---

At a village in America a fine of five dollars is imposed upon persons suffering from diseases of the air passages who expectorate in public places, in or out of doors. It is stated that the prohibition has been respected, as so far it has been unnecessary to impose any fine. In another part of America where fines are demanded for expectoration in public places, a man was heavily fined for non-observance of the regulation.

---

A convalescent home in connection with the Railway Mission at West Hill, St. Leonards, has been opened by Lady Chichester. The building is of red brick with stone dressings. The rooms are lofty and airy. On the ground floor are dining, consulting, and day rooms, library, and offices. On the first floor are the dormitories, with single rooms for station masters, inspectors, and upper officials. The nurses' and domestic quarters are cut off from the other apartments. In the front of the building is a covered terrace. About £7,700 has already been expended, and a further £1,500 was asked for at the opening ceremony.

---

We have no doubt that many medical men and nurses have never heard of the Aged Pilgrims' Friend Society. Yet the cases are many whom one would gladly see participators in its charity. Alderman Sir Joseph Savory, Bart., M.P., presiding at the annual meeting at the Mansion House on May 3rd, said that poverty, old age, and vital religion were the three qualifications of candidates, and on these lines the work was undenominational. The yearly pensions granted were respectively five guineas, seven guineas, and ten guineas, and during the past year the sum of £10,460 had been distributed to the 1,420 pensioners dependent on the charity. This is a society which many will be glad to know of.

